# Hormonal Adaptations in Professional Soccer Players: Ethnic Differences and Pathophysiological Mechanisms

**DOI:** 10.3390/ijms27125574

**Published:** 2026-06-20

**Authors:** Sandro La Vignera, Rosita A. Condorelli

**Affiliations:** Department of Clinical and Experimental Medicine, University of Catania, Via S. Sofia 78, 95123 Catania, Italy

**Keywords:** testosterone, cortisol, growth hormone, IGF-1, overtraining syndrome, ethnic differences, HPA axis, HPG axis, professional soccer, UGT2B17

## Abstract

Professional soccer imposes substantial physiological demands eliciting complex neuroendocrine responses. This review synthesizes evidence on hormonal adaptations in professional soccer players, with emphasis on ethnic and national differences and underlying pathophysiological mechanisms. We analyzed 21 key studies investigating testosterone, cortisol, the testosterone-to-cortisol (T:C) ratio, growth hormone (GH), and insulin-like growth factor-1 (IGF-1) responses to training and competition. Acute cortisol elevations that may persist for up to 72 h post-match in some professional populations and T:C ratio reductions following congested fixture periods are reported across available studies, while somatotropic responses vary considerably across studies. Preliminary evidence suggests that ethnic and geographic background may influence circulating testosterone and urinary steroid excretion profiles, with UGT2B17 genetic polymorphisms identified as one contributing factor; however, the evidence base is limited and requires replication. Approximately 7.4% of elite junior cohorts—though not necessarily professional adult populations—develop non-functional overreaching (NFOR), characterized by blunted GH and ACTH responses. Pathophysiological mechanisms involve hypothalamic–pituitary––adrenal (HPA) and hypothalamic–pituitary–gonadal (HPG) axis dysregulation producing anabolic–catabolic imbalance. Individualized, longitudinal T:C monitoring and post-match load moderation may be warranted; future research should establish ethnicity-specific normative values and investigate links between hormonal dysregulation and injury risk.

## 1. Introduction

Professional soccer represents one of the most physically and psychologically demanding team sports worldwide, characterized by intermittent high-intensity efforts, frequent competitive matches, and congested fixture schedules that challenge athletes’ adaptive capacity [1,2]. The neuroendocrine system plays a pivotal role in mediating physiological responses to training and competition, orchestrating metabolic, immunological, and neuromuscular adaptations essential for performance optimization and recovery [3,4].

The hypothalamic–pituitary–adrenal (HPA) axis, primarily through cortisol secretion, coordinates the stress response to physical and psychological demands, while the hypothalamic–pituitary–gonadal (HPG) axis, through testosterone production, regulates anabolic processes critical for muscle hypertrophy, recovery, and long-term athletic adaptation [5,6]. The balance between these two axes—quantified as the testosterone-to-cortisol (T:C) ratio—is widely employed in athlete monitoring as an indicator of anabolic–catabolic balance; however, its predictive value for overreaching, overtraining, and performance outcomes remains inconsistent across studies and should be interpreted in conjunction with other clinical and functional indicators [7,8].

A critical yet underexplored dimension of this field concerns ethnic and national differences in hormonal profiles among professional soccer players. It is important to distinguish between ‘ethnic differences’—which may reflect genetic ancestry, including polymorphisms in steroid-metabolizing enzymes distributed differently across populations of different geographic origins—and ‘national differences,’ which may reflect environmental, nutritional, training culture, socioeconomic, and cultural factors varying across countries of origin. Both constructs are addressed together in this review because available studies often report them jointly without fully disentangling their respective contributions, a limitation acknowledged throughout. Contemporary professional soccer teams are increasingly multinational, with players of diverse ethnic backgrounds competing alongside each other. Although players may share the same club training environment and competitive schedule, substantial within-club variability may persist in nutritional habits, cultural practices, recovery behaviors, socioeconomic background, previous training history, and genetic characteristics—all of which may independently influence hormonal profiles. Emerging evidence suggests that ethnic and geographic background may influence baseline steroid concentrations and steroid metabolism, with potential implications for athlete monitoring, anti-doping interpretation, and clinical management; however, the evidence base remains limited and requires replication in larger, multi-center studies [9,10].

This review provides a comprehensive synthesis of the literature on hormonal adaptations in professional soccer players, with particular emphasis on: (1) acute and chronic responses of key hormones to training and competition; (2) ethnic and national differences in hormonal profiles; (3) epidemiological patterns of hormonal dysregulation and overreaching; and (4) pathophysiological mechanisms linking hormonal imbalances to performance outcomes. The review focuses on testosterone, cortisol, GH, and IGF-1 because these represent the hormonal axes with the largest body of published evidence in professional soccer populations and are most directly implicated in the anabolic–catabolic balance relevant to performance, recovery, and overreaching risk. Other endocrine markers—including catecholamines, DHEA-S, prolactin, thyroid hormones, and inflammatory mediators—are also relevant to training adaptation but are not addressed in detail in this review due to the limited soccer-specific evidence base; their exclusion is acknowledged as a limitation.

## 2. Results

### 2.1. Methodological Considerations in Hormonal Assessment

Interpretation of hormonal data in professional soccer requires careful consideration of methodological factors that substantially influence measured values and limit cross-study comparisons. Salivary sampling offers practical advantages in field settings (non-invasive, stress-free collection) and correlates well with serum free cortisol (r = 0.71–0.93) and testosterone; however, salivary values are generally lower than serum concentrations and may be affected by oral contamination, flow rate, and collection timing. Serum/plasma sampling provides higher sensitivity and specificity but requires venepuncture, which may itself elicit stress responses.

Circadian variation critically affects cortisol interpretation: cortisol peaks at approximately 08:00–09:00 h and reaches its nadir around midnight. Sampling must therefore be standardized relative to time of day and time elapsed since waking. GH secretion is pulsatile, with the largest pulse occurring during slow-wave sleep; resting daytime GH concentrations are therefore often undetectable, and exercise-stimulated GH responses provide more reliable information about somatotropic reserve. IGF-1, with its longer half-life, provides a more stable index of chronic GH activity.

Assay methodology also influences comparability: immunoassay-based methods (ELISA, RIA, chemiluminescence) and mass spectrometry-based methods yield different absolute values and have different sensitivity and specificity profiles. The heterogeneity of sampling methods, assay techniques, sampling timing, and load quantification methods across the 21 studies included in this review substantially limits direct quantitative comparisons and must be considered when interpreting synthesized findings.

### 2.2. Testosterone Responses to Training and Competition

Testosterone responses in professional soccer players are heterogeneous, reflecting the complex interplay between training load, competition phase, player position, and individual characteristics. Acute testosterone responses to match play vary considerably across studies, with some reporting modest post-match increases and others documenting significant reductions, particularly following high-intensity or prolonged competitive periods [1,11].

Longitudinal monitoring across competitive seasons reveals more consistent patterns. Di Blasio et al. [6] documented progressive testosterone alterations across a full competitive season, with significant within-season fluctuations correlating with training load and competitive phase. Handziski et al. [7] demonstrated significant reductions in testosterone concentrations during a competition half-season, accompanied by corresponding alterations in adrenocorticotropic hormone (ACTH) and cortisol. Kraemer et al. [11] reported that starters and non-starters exhibited differential hormonal trajectories across a season, with starters showing greater end-of-season testosterone reductions, suggesting cumulative load effects on gonadal function.

Player position may be associated with differential testosterone profiles, as positional differences in running demands and physical contact could theoretically contribute to hormonal variation. Rodríguez-García et al. [12] demonstrated significant associations between testosterone concentrations and anthropometric characteristics in professional male soccer players, suggesting that body composition and hormonal profiles are interrelated. However, these findings represent anthropometric–hormonal associations rather than direct evidence of position-specific hormonal adaptations; direct evidence for position-dependent hormonal profiles in professional soccer remains limited and warrants dedicated investigation.

### 2.3. Cortisol Responses: Acute and Chronic Patterns

Cortisol responses to match play and training are among the most consistently documented endocrine findings in professional soccer research. Match play elicits substantial acute cortisol elevations that may persist for up to 72 h post-match in some professional populations, though the duration and magnitude of this response are likely influenced by competition level, match demands, recovery strategies, and individual player characteristics [5,13]. The 72 h recovery timeframe derives primarily from Springham and Dunbar [5] in an elite professional cohort and should not be assumed to be universally applicable across all professional soccer contexts.

Springham et al. [2] demonstrated in a longitudinal analysis that summated training and match loads were significant predictors of salivary cortisol changes in elite professional football players, with non-linear relationships between chronic high-intensity running loads and cortisol profiles. Rowell et al. [1] reported that a 1 SD acute load increase was associated with reduced T:C balance in positional subgroups, highlighting the sensitivity of cortisol to acute training stimuli.

Seasonal cortisol dynamics reveal progressive elevations across competitive phases, with preseason periods characterized by both testosterone and cortisol increases in some cohorts [6,12]. Filaire et al. [3] documented biological, hormonal, and psychological parameter changes throughout a competitive season, establishing that cortisol trajectories are closely linked to competitive schedule density and psychological stress.

The COVID-19 pandemic provided a unique natural experiment for examining hormonal responses to detraining. Muscella et al. [14] reported significant alterations in biological parameters including hormonal profiles during lockdown-induced training cessation, with subsequent rebound effects upon return to training, underscoring the dynamic nature of hormonal adaptation in professional soccer players.

### 2.4. Testosterone-to-Cortisol Ratio as a Biomarker of Training Adaptation

The T:C ratio has been proposed as an integrated indicator reflecting the anabolic–catabolic hormonal milieu. Values below population-based reference ranges have been associated with impaired recovery and increased overreaching risk in some studies, though the precise cutoff remains debated and no universally accepted threshold has been established [15].

Banfi and Dolci [15] proposed a practical categorization of free T:C ratio values within professional soccer squads, suggesting potential utility of longitudinal tracking for identifying players at risk of hormonal imbalance. Their categorization approach—distinguishing optimal, borderline, and at-risk zones—has been adopted in several subsequent monitoring protocols, though its external validity across different populations and measurement methodologies has not been systematically validated.

Saidi et al. [8] provided evidence for the sensitivity of T:C ratio to fixture congestion, demonstrating that a congested match period was associated with significantly lower testosterone and T:C values, accompanied by worse vigor/fatigue scores and objective decrements in jump and intermittent fitness tests. Correlations between workload metrics and hormonal changes are consistent with the potential utility of T:C ratio as a load monitoring indicator, though causal relationships cannot be established from observational data.

Rowell et al. [1] extended these findings through a season-long investigation, demonstrating that neuromuscular recovery, testosterone, and cortisol profiles were significantly influenced by both training and competition load, with T:C ratio providing possible additional information for performance outcome assessment beyond individual hormone measurements. However, the clinical utility of the T:C ratio must be interpreted in light of its substantial intra- and inter-individual biological variability, sensitivity to sampling methodology (salivary vs. serum; free vs. total testosterone), and inconsistent predictive value for overreaching and performance outcomes reported across studies. The T:C ratio should therefore be considered one component of a multi-parameter monitoring approach rather than a standalone diagnostic tool.

### 2.5. Growth Hormone and IGF-1: Somatotropic Axis Responses

The somatotropic axis—comprising GH and its primary mediator IGF-1—plays essential roles in tissue repair, muscle hypertrophy, and metabolic regulation. However, somatotropic responses to training and competition in professional soccer players are inconsistent across studies, reflecting methodological heterogeneity and the pulsatile nature of GH secretion [16]. It should be noted that much of the available evidence on somatotropic responses in soccer derives from youth or developing players rather than professional adult populations, and findings should not be directly extrapolated to professional adults without appropriate qualification.

Hadjicharalambous [16] reported no consistent change in resting GH or IGF-1 following preseason preparation blocks in young high-level soccer players (not professional adults), suggesting that acute training stimuli may be insufficient to produce lasting alterations in basal somatotropic activity in developing athletes. Hammami et al. [17,18] documented context-dependent somatotropic responses over two-year monitoring periods in elite young soccer players, with hormonal changes closely tracking training phase and physical fitness development. The applicability of these findings to professional adult soccer players requires further investigation.

In contrast, states of non-functional overreaching are consistently associated with somatotropic dysregulation. Schmikli et al. [13] demonstrated that players with sustained performance decreases meeting NFOR criteria showed reduced resting GH and blunted ACTH responses to maximal exercise, alongside mood disturbances, indicating hypothalamic-pituitary axis dysfunction in overreached states. This finding has important diagnostic implications, as attenuated GH responses to standardized exercise stimuli may serve as objective biomarkers of NFOR.

### 2.6. Ethnic and National Differences in Hormonal Profiles

It is important to note at the outset that the evidence on ethnic differences in hormonal profiles among professional soccer players is currently limited to a small number of studies, and findings should therefore be considered preliminary pending replication in larger, multi-center cohorts with adequate control for potential confounders including diet, supplementation, training load, socioeconomic status, and cultural practices.

#### 2.6.1. Circulating Hormonal Differences

**Figure 1:**
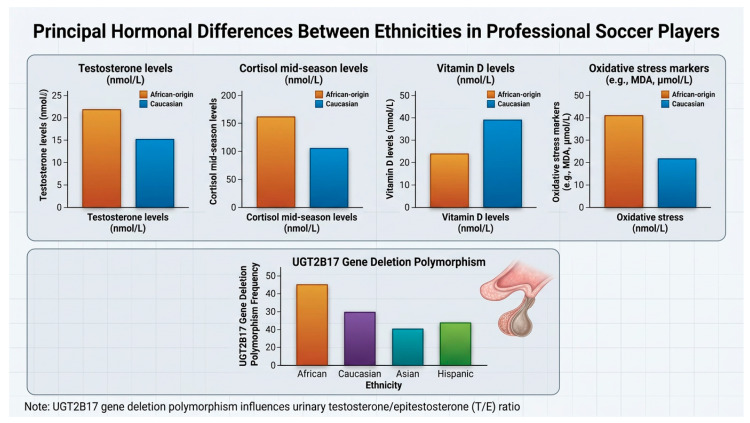
Principal hormonal differences between ethnicities in professional soccer players. Bar charts compare testosterone, cortisol mid-season, vitamin D, and oxidative stress marker (MDA) levels between African-origin (orange) and Caucasian (blue) players. Lower panel: UGT2B17 gene deletion polymorphism frequency by ethnicity. Data adapted from Abate and Salini [19] and Strahm et al. [9].

Abate and Salini [19] conducted an investigation comparing oxidative stress, testosterone, cortisol, and vitamin D levels between professional soccer players of African and Caucasian geographic origin at pre-season and mid-season time points. African-origin players exhibited higher circulating testosterone concentrations at both sampling points in this study, while mid-season cortisol and oxidative stress markers were also elevated in this group. Conversely, vitamin D levels were lower in African-origin players, attributable to reduced cutaneous synthesis at higher latitudes and potentially confounded by dietary differences. These findings derive from a single study with a limited sample size and must be interpreted with caution; they should not be generalized as established characteristics of all players of African geographic origin (Figure 1).

#### 2.6.2. Urinary Steroid Excretion Profiles and Anti-Doping Implications

Strahm et al. [9] provided an international comparative analysis of urinary steroid profiles in professional soccer players across African, Asian, Caucasian, and Hispanic ethnic groups. Significant inter-ethnic differences in urinary steroid profiles were documented, with a substantial proportion of variability in urinary T/E ratios attributable to the UGT2B17 deletion polymorphism. It is important to distinguish between urinary steroid excretion profiles—which reflect the activity of steroid-glucuronidating enzymes and are relevant primarily to anti-doping applications—and circulating serum/plasma hormonal concentrations, which are relevant to clinical monitoring. These two dimensions are related but not interchangeable, and conclusions drawn from urinary steroid data should not be directly applied to clinical hormonal monitoring strategies.

The practical implications are substantial: ethnicity and genotype materially alter urinary steroid excretion profiles, necessitating longitudinal individual monitoring and genotype-aware thresholds for anti-doping purposes rather than single universal cutoffs [9]. These findings have been incorporated into the World Anti-Doping Agency (WADA) Athlete Biological Passport framework, which employs adaptive Bayesian models that account for individual variation over time.

National differences in hormonal profiles extend beyond ethnicity per se to encompass environmental, nutritional, and training culture factors. Players from different nations may have been exposed to distinct training philosophies, dietary practices, and environmental conditions that influence hormonal baselines and adaptation patterns. However, robust comparative data across national cohorts remain limited in the published literature, and the relative contributions of genetic, environmental, and cultural factors to observed national differences cannot be quantified from the available evidence.

### 2.7. Epidemiological Findings: Overtraining Syndrome and NFOR

Despite the clinical importance of overtraining syndrome (OTS) and its precursor state, non-functional overreaching (NFOR), robust population-level epidemiological data on their prevalence in professional soccer are sparse. Available evidence derives primarily from single-team longitudinal studies and cohort monitoring programs.

Schmikli et al. [13] conducted a prospective cohort study monitoring 94 elite junior soccer players across a competitive season, applying field-based performance criteria combined with hormonal and mood profile assessment to identify players meeting NFOR criteria—representing one of the most comprehensive monitoring designs available in the soccer literature. Seven players (approximately 7.4%) met criteria for sustained performance decrease associated with NFOR-consistent hormonal and mood changes. This estimate derives from an elite junior population and should not be interpreted as a population-level prevalence estimate for professional adult soccer players, in whom training load management, recovery resources, and monitoring practices may differ substantially. Population-level epidemiological data on NFOR in professional adult soccer are currently lacking and represent an important research gap.

Multiple single-team seasonal studies document frequent T:C perturbations, significant within-season testosterone and cortisol alterations, and episodic post-match catabolic states [1,2,3,7]. Handziski et al. [7] reported significant ACTH, cortisol, and testosterone changes across a competition half-season in professional players, consistent with progressive HPA and HPG axis loading. Filaire et al. [3] documented biological, hormonal, and psychological parameter changes throughout a full season, establishing correlations between hormonal perturbations and performance and mood outcomes.

The case report by Naessens et al. [10] illustrates the clinical endpoint of severe hormonal dysregulation: hypogonadism presenting as recurrent muscle injury in a high-level soccer player, underscoring the potential musculoskeletal consequences of chronic hormonal imbalance.

### 2.8. Pathophysiological Mechanisms

#### 2.8.1. HPA Axis Dysregulation

The HPA axis represents the primary neuroendocrine pathway mediating the stress response in professional soccer players. Repeated activation by training and competition leads to cumulative cortisol exposure that, when excessive or unrelieved, is associated with maladaptive changes in HPA axis regulation [5,7].

Based on evidence from the broader exercise endocrinology literature, chronic HPA axis activation has been hypothesized to involve progressive glucocorticoid receptor downregulation, reduced negative feedback sensitivity, and altered diurnal cortisol rhythmicity. These changes manifest clinically as persistently elevated basal cortisol, blunted cortisol responses to novel stressors, and disrupted sleep architecture—features reported in athletes with NFOR and OTS [13]. The hypothalamic CRH system is also sensitive to psychological stressors; however, direct evidence for CRH dysregulation specifically in professional soccer players is currently limited.

#### 2.8.2. HPG Axis Alterations

The HPG axis is subject to inhibitory modulation by chronic cortisol excess through multiple mechanisms. Based on evidence from the broader endocrinology literature, glucocorticoids have been shown to suppress GnRH pulsatility at the hypothalamic level, reduce pituitary sensitivity to GnRH, and directly impair Leydig cell steroidogenesis in experimental models [10,11]; however, the extent to which these mechanisms operate in professional soccer players has not been directly investigated. The net effect is functional hypogonadism—reduced testosterone production—which may present clinically as reduced libido, impaired recovery, and increased injury susceptibility.

Naessens et al. [10] documented this endpoint in a professional soccer player presenting with recurrent muscle injuries, in whom comprehensive endocrine evaluation revealed hypogonadism as the underlying etiology. This case illustrates the potential clinical consequences of chronic HPG axis suppression.

#### 2.8.3. Anabolic–Catabolic Balance

The T:C ratio provides an operational index of the anabolic–catabolic hormonal balance, with reductions below reference ranges associated with a shift toward catabolism that may impair muscle protein synthesis, delay recovery, and increase injury risk in susceptible individuals [15]. The physiological basis for this index lies in the opposing actions of testosterone (promoting muscle protein synthesis, satellite cell activation, and glycogen resynthesis) and cortisol (promoting protein catabolism, gluconeogenesis, and immune modulation) on skeletal muscle [1,8].

Congested fixture periods represent particularly high-risk contexts for anabolic–catabolic imbalance. Saidi et al. [8] demonstrated that a congested match period was associated with significant T:C ratio reductions accompanied by objective performance decrements and mood disturbances, consistent with a mechanistic link between hormonal imbalance and functional outcomes, though causality cannot be established from observational data. The temporal dynamics of T:C recovery following matches—which may require up to 72 h for normalization in some populations [5]—have potential implications for training prescription and fixture scheduling.

#### 2.8.4. Somatotropic Axis and Tissue Repair

GH and IGF-1 play critical roles in tissue repair and muscle hypertrophy, making somatotropic axis dysregulation in NFOR potentially consequential for injury risk and recovery capacity. Schmikli et al. [13] demonstrated blunted GH responses to maximal exercise in overreached junior players, suggesting impaired somatotropic reserve. It has been hypothesized, based on the established roles of GH and IGF-1 in tissue repair and muscle hypertrophy, that this somatotropic dysregulation may compromise recovery from micro-injuries and delay adaptive responses to training stimuli; however, this mechanism has not been directly demonstrated in professional soccer populations and warrants prospective investigation.

The molecular mechanisms underlying somatotropic dysregulation in overreaching are hypothesized to involve hypothalamic GHRH suppression by elevated somatostatin tone and direct inhibitory effects of elevated cortisol on GH secretion and IGF-1 hepatic production [16,17]. These mechanisms derive from broader exercise endocrinology literature and have not been directly demonstrated in professional soccer populations.

## 3. Discussion

The evidence synthesized in this review suggests that professional soccer imposes substantial neuroendocrine demands, producing characteristic hormonal response patterns that vary with training load, competitive phase, player position, and ethnic background. Several key themes emerge with potential practical significance for athlete monitoring and management, though the strength of available evidence must be carefully considered.

First, the T:C ratio represents a potentially useful hormonal indicator in professional soccer, providing an integrated index of anabolic–catabolic balance that has been associated with performance outcomes, mood states, and recovery quality in some studies [1,8,15]. However, its predictive value for overreaching and performance outcomes remains inconsistent across studies, and its utility is subject to substantial intra- and inter-individual biological variability and methodological sensitivity. Its utility is best realized through longitudinal, individualized monitoring that establishes player-specific reference ranges.

Second, preliminary evidence suggests that ethnic and geographic background may influence hormonal profiles and urinary steroid excretion patterns in professional soccer players. The higher baseline testosterone observed in African-origin players in one study [19], combined with ethnic differences in UGT2B17-mediated steroid glucuronidation [9], suggests that ethnicity-aware interpretation of hormonal values and anti-doping steroid ratios may be warranted; however, these findings derive from a very limited number of studies and should not be presented as established clinical guidelines. The establishment of validated, ethnicity-specific reference ranges represents a priority for future research rather than a current clinical recommendation.

Third, the approximately 7.4% prevalence of NFOR reported in one elite junior cohort [13] suggests that hormonal dysregulation may not be uncommon in high-level soccer; however, this estimate should not be generalized to professional adult populations. Population-level epidemiological data on NFOR in professional adult soccer are lacking and represent an important research gap.

Fourth, post-match hormonal perturbations may persist for up to 72 h in some professional populations [5], supporting the principle of load moderation in the immediate post-match window; however, the generalizability of this recommendation across all professional soccer settings requires further investigation, as the magnitude and duration of post-match hormonal responses are likely influenced by competition level, match demands, fixture congestion, recovery strategies, training status, and measurement methodology.

The limitations of this evidence base warrant explicit acknowledgment. Most studies are conducted in single teams or small cohorts, limiting generalizability. Methodological heterogeneity in hormonal sampling (salivary vs. plasma, morning vs. post-exercise), assay techniques, and load quantification methods impedes direct comparison across studies. The evidence base is weighted toward studies published before 2015, and more recent data using contemporary monitoring technologies are needed. Most studies are observational in nature, precluding causal inference. Population-level epidemiological data on endocrine disorders in professional soccer are conspicuously absent. The mechanistic evidence linking specific hormonal patterns to injury outcomes is largely inferential rather than prospective. The review focuses on testosterone, cortisol, GH, and IGF-1; other endocrine markers including catecholamines, DHEA-S, prolactin, thyroid hormones, and inflammatory mediators are not addressed, representing a further limitation.

## 4. Materials and Methods

This review was conducted as a comprehensive narrative literature review. The narrative format was selected to accommodate the methodological heterogeneity of available studies (observational cohorts, cross-sectional designs, case reports, and RCTs with incompatible outcome measures that preclude meta-analytic pooling). Electronic database searches were performed on January 2025 in PubMed/MEDLINE, Google Scholar, and SciSpace, covering studies published from 1995 to 2024 (no date restriction applied). The following search terms and Boolean combinations were used: (“soccer” OR “football”) AND (“testosterone” OR “cortisol” OR “growth hormone” OR “IGF-1” OR “hormonal adaptations” OR “endocrine responses” OR “steroid hormones”) AND (“professional” OR “elite” OR “athlete”); (“soccer” OR “football”) AND (“ethnic” OR “racial” OR “national” OR “ancestry” OR “UGT2B17” OR “genetic polymorphism” OR “African” OR “Caucasian” OR “Asian”) AND (“hormonal” OR “testosterone” OR “cortisol”); (“soccer” OR “football”) AND (“overtraining” OR “overtraining syndrome” OR “non-functional overreaching” OR “endocrine disruption” OR “athlete monitoring” OR “recovery” OR “training load”) AND (“epidemiology” OR “pathophysiology” OR “injury”).

**Figure 2:**
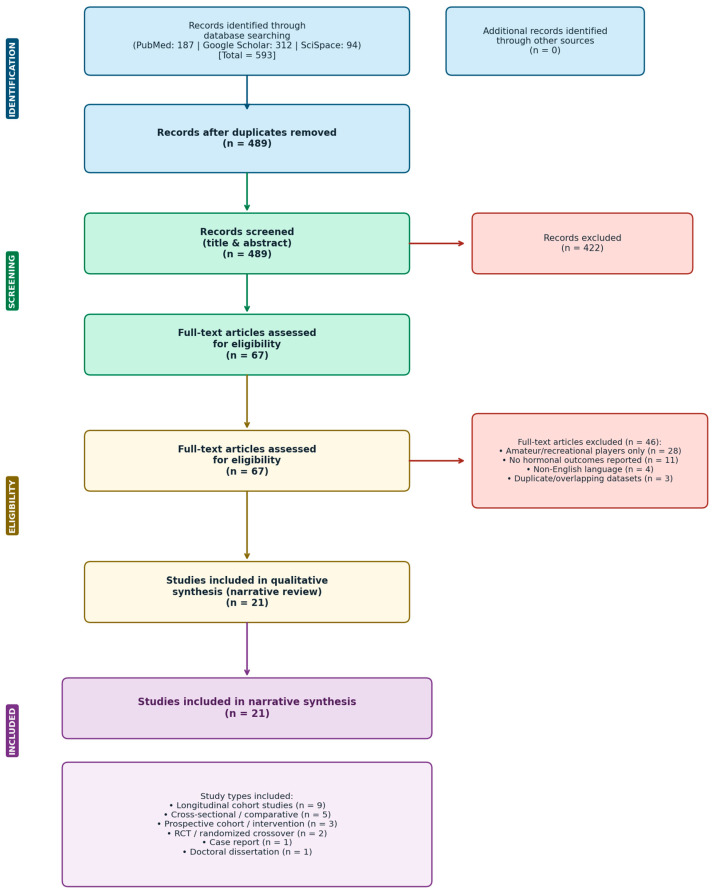
PRISMA-style flow diagram of the study selection process. Records were identified from three databases (PubMed/MEDLINE: 187; Google Scholar: 312; SciSpace: 94). After deduplication (*n* = 489), title/abstract screening (*n* = 489→67), and full-text review (*n* = 67→21), 21 studies were included in the narrative synthesis.

The literature search yielded the following records: PubMed/MEDLINE: 187 records; Google Scholar: 312 records (first 100 results per search string screened by relevance); SciSpace: 94 records; total records identified: 593; after deduplication: 489 records. Records retrieved from Google Scholar and SciSpace were screened using the same criteria applied to PubMed/MEDLINE records. After title and abstract screening: 67 potentially eligible studies. After full-text review: 21 studies included. Reasons for exclusion at full-text review: amateur/recreational populations only (*n* = 28); no hormonal outcomes reported (*n* = 11); non-English language (*n* = 4); duplicate/overlapping datasets (*n* = 3). Study selection was performed independently by both authors; discrepancies were resolved by consensus discussion. A PRISMA-style flow diagram describing the selection process is presented in Figure 2.

Studies were included if they: (1) investigated hormonal responses in soccer/football players; (2) included professional or elite-level participants; (3) measured at least one hormonal outcome (testosterone, cortisol, GH, IGF-1, or ACTH); and (4) were published in peer-reviewed journals or presented as doctoral dissertations. Studies were excluded if they examined amateur or recreational players exclusively, did not report hormonal outcomes, or were published in languages other than English.

The single doctoral dissertation included (Hadjicharalambous 2010) [16] was retained because it represents one of the few available investigations of somatotropic responses to preseason training in soccer players; its methodological quality was assessed using the same domain-based criteria applied to peer-reviewed studies (Table 1). The 21 included studies represent all studies meeting the pre-specified eligibility criteria after the full two-stage screening process. A supplementary search using additional ethnicity-related terms (‘ethnicity,’ ‘race,’ ‘ancestry,’ ‘UGT2B17,’ ‘genetic polymorphism,’ ‘African,’ ‘Caucasian,’ ‘Asian’) confirmed the limited evidence base, yielding only the two studies already included.

## 5. Conclusions

Professional soccer imposes complex neuroendocrine demands that produce characteristic hormonal adaptation patterns with important implications for performance, recovery, and health. Among the hormonal biomarkers reviewed, the T:C ratio represents a potentially useful indicator for monitoring training adaptation and anabolic–catabolic balance; however, its predictive value for overreaching and performance outcomes remains inconsistent across studies, and its clinical utility should be evaluated in conjunction with other functional and psychological indicators. Standardization of measurement methodology and establishment of population-specific reference ranges are needed before the T:C ratio can be recommended as a routine clinical monitoring tool. Preliminary evidence suggests that ethnic and geographic background may influence baseline steroid concentrations and urinary steroid excretion profiles in professional soccer players; however, the evidence base is currently limited to a small number of studies, and the establishment of validated, ethnicity-specific reference ranges for hormonal monitoring represents an important priority for future research rather than a current clinical recommendation. The approximately 7.4% prevalence of NFOR reported in one elite junior cohort underscores the potential frequency of hormonal dysregulation in high-level soccer; however, this estimate should not be generalized to professional adult populations, and population-level epidemiological data on NFOR in professional adult soccer are lacking. Available evidence from a limited number of professional soccer studies suggests that post-match hormonal perturbations may persist for up to 72 h in some populations, supporting the principle of load moderation in the immediate post-match window; however, the generalizability of this recommendation requires further investigation, as post-match hormonal responses are likely influenced by competition level, match demands, fixture congestion, recovery strategies, training status, and measurement methodology. Future research priorities include: (1) establishing standardized, ethnicity-specific normative values for key hormonal biomarkers in professional soccer players; (2) prospective investigation of mechanistic links between hormonal dysregulation and musculoskeletal injury risk; (3) population-level epidemiological studies on endocrine disorders across professional leagues and nations; and (4) development of validated, practical monitoring protocols integrating hormonal, neuromuscular, and psychological biomarkers for individualized athlete management.

## Figures and Tables

**Table 1:** Quality Assessment column added (Newcastle-Ottawa Scale/Cochrane RoB).

Authors (Year)	Study Design	Population	Country/Ethnicity	Hormones Assessed	Key Findings	Quality Assessment (Risk of Bias)
Rowell et al. (2018) [1]	Longitudinal cohort	18 professional male players	Australia	T, C, T:C ratio	1 SD acute load increase reduced T:C in positional subgroups; neuromuscular recovery correlated with hormonal profiles	*Low RoB* *(prospective cohort, n = 18, objective load metrics)*
Springham et al. (2021) [2]	Longitudinal cohort	Elite professional male players	UK	sIgA, α-amylase, T, C, T:C	Summated training/match loads predict salivary T and C changes; non-linear dose–response for chronic high-intensity running	*Low RoB* *(longitudinal, large n, validated load measures)*
Filaire et al. (2003) [3]	Longitudinal cohort	15 professional male players	France	T, C, T:C, psychological markers	Progressive cortisol elevations across season; T:C correlated with mood and performance outcomes	*Moderate RoB* *(small n = 15, no control group)*
Abate & Salini (2022) [19]	Cross-sectional comparative	Professional male players	Italy (African vs. Caucasian)	T, C, Vitamin D, oxidative stress	African-origin players: higher T and cortisol, lower Vitamin D, higher oxidative stress markers	*Moderate RoB* *(cross-sectional, limited confounders control)*
Strahm et al. (2009) [9]	International cross-sectional	Professional male players	Multi-national (4 ethnicities)	Urinary steroid profile, T/E ratio	Significant ethnic differences in urinary steroid profiles; UGT2B17 polymorphism explains T/E variability	*Moderate RoB* *(cross-sectional, multi-ethnic, limited n)*
Schmikli et al. (2012) [13]	Prospective cohort	94 elite junior male players	The Netherlands	GH, ACTH, mood, performance	NFOR prevalence ~7.4%; blunted GH and ACTH responses in overreached players	*Low RoB* *(prospective cohort, n = 94, standardized criteria)*
Handziski et al. (2006) [7]	Longitudinal cohort	Professional male players	North Macedonia	ACTH, C, T, T:C	Significant ACTH, C, T reductions across competition half-season; T:C declined progressively	*Moderate RoB* *(no control group, limited reporting)*
Saidi et al. (2020) [8]	Prospective intervention	Professional male players	Tunisia	T, C, T:C, fitness markers	Congested match period: lower T and T:C, worse vigor/fatigue scores, decrements in jump and fitness tests	*Low RoB* *(prospective, validated fitness measures)*
Di Blasio et al. (2015) [6]	Longitudinal cohort	Male soccer players	Italy	C, T, T:C	Progressive cortisol rise across season; seasonal T:C fluctuations correlated with training load	*Moderate RoB* *(longitudinal cohort, limited n)*
Kraemer et al. (2004) [11]	Longitudinal cohort	26 collegiate male players	USA	T, C, T:C, performance	Starters showed greater end-of-season T reductions; low T/high C associated with performance decline	*Moderate RoB* *(longitudinal cohort, 26 players)*
Hadjicharalambous (2010) [16]	Pre-post intervention	Young high-level male players	Greece	GH, IGF-1, T, C	No consistent resting GH/IGF-1 changes after preseason; acute training insufficient for lasting somatotropic alterations	*High RoB* *(doctoral dissertation, not peer-reviewed)*
Hammami et al. (2017) [17]	2-year longitudinal	Elite young male players	Tunisia	T, C, cortisol-gonadotropic axis	Progressive hormonal changes over 2-year training; cortical-gonadotropic axis adaptations correlated with physical fitness	*Moderate RoB* *(2-year longitudinal, youth population)*
Hammami et al. (2018) [18]	2-year longitudinal	Elite young male players	Tunisia	T, C, somatotype hormones	Somatotype and hormonal profiles co-evolved with physical fitness development across 2-year monitoring	*Moderate RoB* *(2-year longitudinal, youth population)*
Banfi & Dolci (2006) [15]	Cross-sectional	Professional male players	Italy	Free T:C ratio	Proposed practical categorization of free T:C values; longitudinal tracking identifies at-risk players	*Moderate RoB* *(cross-sectional, professional players)*
Springham & Dunbar (2022) [5]	Prospective cohort	Elite professional male players	UK	Immunological and hormonal markers	Match-induced hormonal perturbations persist ≥72 h; post-match load moderation recommended	*Low RoB* *(prospective cohort, elite professional)*
Rodríguez-García et al. (2024) [12]	Cross-sectional	Professional male players	Spain	T, C, anthropometrics	Significant T and C correlations with body composition; position-dependent hormonal profiles	*Moderate RoB* *(cross-sectional, anthropometric focus)*
Muscella et al. (2023) [14]	Pre-post natural experiment	Professional male players	Italy	Biological parameters, hormones	COVID-19 lockdown altered hormonal profiles; rebound effects upon return to training	*Moderate RoB* *(natural experiment, COVID context)*
Enright et al. (2017) [20]	Randomized crossover	Youth elite male players	UK	T, C, T:C	Training organization influences hormonal responses to concurrent exercise in youth players	*Low RoB* *(RCT crossover, youth elite)*
Naessens et al. (1995) [10]	Case report	1 high-level male player	Belgium	T, LH, FSH	Hypogonadism as cause of recurrent muscle injury; endocrine evaluation essential in refractory cases	*High RoB* *(case report, n = 1)*
Hammami et al. (2017) [17]	Longitudinal	Elite young male players	Tunisia	T, C, GH, IGF-1	Hormonal adaptations track physical performance across training phases	*Moderate RoB* *(longitudinal, youth population)*
Gorostiaga et al. (2004) [21]	RCT	Young male soccer players	Spain	T, C, GH, IGF-1	Strength training produced performance improvements with hormonal adaptations; T and GH increased post-intervention	*Low RoB* *(RCT, young players, controlled design)*

T, testosterone; C, cortisol; T:C, testosterone-to-cortisol ratio; GH, growth hormone; IGF-1, insulin-like growth factor-1; sIgA, secretory immunoglobulin A; ACTH, adrenocorticotropic hormone; LH, luteinizing hormone; FSH, follicle-stimulating hormone; NFOR, non-functional overreaching; T/E, testosterone/epitestosterone; RoB, Risk of Bias.

## Data Availability

No new data were created or analyzed in this study. The original contributions presented in this study are included in the article. Further inquiries can be directed to the corresponding author.
